# Assessing Relevance of External Cognitive Measures

**DOI:** 10.3389/fnint.2017.00003

**Published:** 2017-02-21

**Authors:** Osvaldo Cairó

**Affiliations:** Department of Computer Science, Instituto Tecnológico Autónomo de México (ITAM)Mexico City, Mexico

**Keywords:** brain, brain network, brain tissue components, brain plasticity, cognition, MRI scan IQ, environmental factors

## Abstract

The arrival of modern brain imaging technologies has provided new opportunities for examining the biological essence of human intelligence as well as the relationship between brain size and cognition. Thanks to these advances, we can now state that the relationship between brain size and intelligence has never been well understood. This view is supported by findings showing that cognition is correlated more with brain tissues than sheer brain size. The complexity of cellular and molecular organization of neural connections actually determines the computational capacity of the brain. In this review article, we determine that while genotypes are responsible for defining the theoretical limits of intelligence, what is primarily responsible for determining whether those limits are reached or exceeded is experience (environmental influence). Therefore, we contend that the gene-environment interplay defines the intelligent quotient of an individual.

## Introduction

People have long been tempted to link brain size and intelligence (Morton, 1839; Broca, 1861; Galton, 1889; Terman, 1926; Sorokin, 1927; Hooton, 1939). Broca (1861) stated many years ago that brain size is directly related to intellectual achievement. “He observed that skilled workers and individuals prominent in their fields had larger brains than those who had not achieved such distinction” (Cairó, 2011). Terman (1926) further demonstrated that, on an average, individuals with special talents had larger brains than those who did not.

In recent years, the advent of modern brain imaging technologies has provided new opportunities for examining the biological essence of human intelligence (Luders et al., 2009), as well as the relationship between brain size and cognition. Thanks to the advances in science and technology, most recent studies show on the contrary that the relationship between brain size and intelligence has never been well understood. This view is supported by findings showing that general intellectual ability is correlated more with the density of neurons, the isocortex, the cerebellum, the forebrain and gray matter volume (which is dense in neural cell bodies) in the frontal lobe, parietal lobe and Broca’s area than sheer brain size (Bracke-Tolkmitt et al., 1989; Krugger et al., 2003; Rogers, 2004; Roth and Dicke, 2005; Colom et al., 2006; Jung and Haier, 2007; Neubauer and Fink, 2009; Langer et al., 2011). Notwithstanding these findings, “neuroscientists now believe that the complexity of cellular and molecular organization of neural connections is what truly determines a brain’s computational capacity” (Lechtenberg, 2014).

There are two categories of brain measures in the brain size literature. The first one consists of measures of the external size of the head, such as the circumference of the head (Gignac et al., 2003; Ivanovic et al., 2004; Luders et al., 2009; Rushton and Ankney, 2009). The second one is much more interesting and is related to the brain volume, which can be assessed using techniques such as magnetic resonance imaging (MRI), functional MRI (fMRI), positron emission tomography (PET) and other non-invasive measures of brain structure and activity (Willerman et al., 1991; Andreasen et al., 1993; Wickett et al., 2000; Videbech and Ravnkilde, 2004; McDaniel, 2005; Witelson et al., 2006; Pietschnig et al., 2015).

The idea behind these studies can be summarized in the following way: the larger the brain, the higher the IQ. “This assumption is substantiated by three facts: (a) an increase in size provides an increase in function; (b) much of the brain is occupied by cortical associations; and (c) the cerebral cortex encompasses more than two-thirds of the brain mass and it is responsible for thinking, perceiving, producing and understanding language. Therefore, if the brain is larger, then the cerebral cortex must be bigger” (Cairó, 2011).

Other studies suggest the same: large brains are a result of differential increases in size of parts of the brain according to their importance (Mace et al., 1980); brain size is proportional to its capacity for processing information (Jerison, 1985); larger brains have more neurons (Haug, 1987); the correlation between brain size and number of neurons is 0.56 (Pakkenberg and Gundersen, 1997); and an increase in brain size implies an increase in conceptual or semantic complexity (Gibson, 2002).

All of these assumptions seem logical. However, there are a number of substantial problems with some of the assumptions that support the hypotheses. Some cetaceans, such as sperm whales and elephants, have much bigger brains than humans (their brain size can exceed 8 kg) and other animals, but they are not smarter. There are also individuals with large brains and low intelligence levels, such as those with an abnormal accumulation of cerebrospinal fluid in the ventricles (hydrocephalus). On the other hand, there are individuals with a small brain and a tremendous intelligence level, which somewhat contradicts the above hypothesis. The cranial capacity of Einstein was 1230 cm^3^, considerably less than the average size for an adult (Anderson and Harvey, 1996).

The volume of the human brain reached its peak up to 1600 cm^3^ in Homo neanderthalensis, but it has been getting smaller over the past 28,000 years (Henneberg, 1988). Domestic animals also have smaller brains than their wild ancestors. In summary, most of the studies report weak correlations from 0.17 to 0.26 between external head size measures and intelligence (van Valen, 1974; Susanne, 1979; Nguyen and McDaniel, 2000; Vernon et al., 2000). Head dimensions clearly do not explain very much of the variation in IQ.

Studies evaluating the correlation between brain volume and intelligence are much more interesting, but they are scarce. In a large meta-analysis consisting of 88 studies, Pietschnig et al. (2015) estimated the correlation between brain volume and intelligence to be about 0.24. McDaniel (2005) reported this correlation in 0.33. Most studies, however, conclude that the correlation between IQ and brain volume is consistently in the 0.3–0.4 range (Egan et al., 1994; Nguyen and McDaniel, 2000; Vernon et al., 2000; Witelson et al., 2006; Taki et al., 2012).

### Tools for Comparing Encephalization within Species

Brain size tends to vary according to body size. Therefore, there is a good possibility that a power law, such as the brain-to-body weight ratio (or brain-to-body height), exists. This law may well become a useful tool for comparing encephalization within species. This by one means that relative instead of absolute brain size coincides much better with observed cognitive abilities in animals. The human male ratio is 1.86%, the bottle-nosed dolphin ratio is 1.25%, and the chimpanzee ratio is 0.4%. The results tend to substantiate to some degree the belief that the humans are the smartest and the most evolved in the animal kingdom. However, it has also some inconsistencies. A shrew’s brain weighs 3 g and its total weight is 30 g, making its ratio 10%. This tiny animal has the highest brain-to-body mass ratio of any known creature.

### Encephalization Quotient (EQ)

A sophisticated alternative to external measures of cognition is the encephalization quotient (EQ) that takes into account allometric effects of widely divergent body sizes. “For a larger organism, more brain capacity is needed to perform basic survival tasks, such as thermoregulation, breathing and motor skills. As the brain gets larger in relation to body size, it will have greater available capacity for performing cognitive tasks” (Cairó, 2011).

The EQ formula was developed for Jerison (1973) in the late 1960s and is calculated as follows:

$$\text{EQ}\;=\;\text{brain-weight}/(0.12*{\text{body-weight}}^{\left(2/3\right)})$$

The constants were derived empirically for mammals and represent an important geometric relationship between volume and surface area. The exponential constant for primates is 0.28 (Williams, 2002). Martin (1981) and Savage et al. (2004) estimated the power of 3/4 for some of the other vertebrate classes based in a large set of cases. The formula may give no meaningful results for invertebrates, because as Stephen Gould says “they do not have spinal cords or, in some cases, central nervous systems”.

The average EQ is defined as 1. The tiger reaches an EQ of 0.68; the whale dolphin an EQ of 5.55; and a human being an EQ of 6.54. Russell (1983) found that the EQ of a mammal that lived 65 million years ago was 0.30, compared to the average of 1.0 today.

The EQ formula can reasonably be seen as a good predictor of intelligence in mammals, but it has some shortcomings. For instance, it does not consider the fact that completely distorts the formula and others such as density of neurons, cortical thickness, number of cortical neurons, and brains folding that are very important and may correlate better with human cognition (Cairó, 2011).

### Brain Size and Intelligence: Does the Size Matter?

The relationship between brain size and intelligence is controversial. Many articles support one point of view, while many others refute it. Both sides substantiate their position with arguments and disqualify the opposing view. As opposed to bolster either position, we underscore a third view that has gained acknowledgment and credibility in recent years: genes and environment cannot be assessed separately.

## Brain Parts, Brain Tissue Components and Brain Network

Researchers have been trying to pinpoint the substrate of human intelligence for decades. Thanks to the advances in science and technology, most recent studies now show that the relationship between brain size and intelligence has never been well understood. This view is supported by findings showing that intelligence is correlated with more subtle characteristics such as density of neurons, the isocortex, the cerebellum, the forebrain, white matter and gray matter volume in the frontal lobe, parietal lobe and Broca’s area than sheer brain size (Bracke-Tolkmitt et al., 1989; Krugger et al., 2003; Rogers, 2004; Roth and Dicke, 2005; Colom et al., 2006; Jung and Haier, 2007; Neubauer and Fink, 2009; Langer et al., 2011). Even the speed at which nerve impulses travel in the brain could be linked to intelligence. Despite these findings, recent studies conclude that the complexity of cellular and molecular organization of neural connections actually determines the computational capacity of the brain (Barbey et al., 2012; Cole et al., 2012; Lechtenberg, 2014; Vakhtin et al., 2014; Bohlken et al., 2016). The consistency of these new results across the literature has revealed that individuals with more efficient brain networks have a higher intelligence.

The brain contains three tissue types: gray matter, white matter and cerebrospinal fluid. Luders et al. (2009) say that quantifying the amount of gray matter gives an estimate of the density and number of the brain’s neuronal cell bodies and dendritic expansions; quantifying white matter helps to approximate the number of axons and their degree of myelination. That is, while the amount of gray matter might reflect the capacity of cognition and processing, the amount of white matter might mirror the efficiency of inter-neuronal communication between different brain regions. “White matter of a 20-year-old man contains a staggering 176,000 km of myelinated axons” (Marner et al., 2003). “Axons ensure smooth communication throughout the brain by conducting electrical impulses and by transporting various molecules and organelles from the cell body to a synapse” (Barry et al., 2007). Gray matter is present in the brain, brainstem and cerebellum, and throughout the spinal cord. On the other hand, white matter forms the bulk of the deep parts of the brain and the superficial parts of the spinal cord.

Recently, Bohlken et al. (2016) posit that “intelligence is associated with a network of distributed gray matter areas including the frontal and parietal higher association cortices and primary processing areas of the temporal and occipital lobes”. Narr et al. (2007) also examined full-scale intelligence quotient (FSIQ) associations with cortical thickness at high spatial resolution across the cortex in 30 male (mean age: 27.9) and 35 female (mean age: 28.5) healthy individuals. To assess general intellectual ability, they employed the Wechsler adult intelligence scale (Wechsler, 1981). “Positive relationships were found between FSIQ and intracranial gray and white matter but not cerebrospinal fluid volumes. Significant associations with cortical thickness were evident bilaterally in prefrontal (Brodmann’s areas [BAs] 10/1147) and posterior temporal cortices (BA 36/37) and proximal regions” (Narr et al., 2007). Haier et al. (2004) have also shown that variation in structures throughout the brain was related to intelligence, finding that there are significant associations between brain variations in gray matter density across discrete areas of the frontal, parietal, temporal and occipital lobes, and IQ scores (Johnson et al., 2008; Colom et al., 2009). Haier et al. (2004) also found that people with high IQ scores had significantly more gray matter in 24 of the regions than people with lower scores. They used voxel-based morphometry (VBM) and a statistical conjunction approach (Price and Friston, 1997) to show where correlations between IQ and gray or white matter were common. “The conjunction results of two samples (*N* = 47) showed robust positive correlations (*P* < 0.05, corrected for multiple comparisons) between FSIQ and gray matter volumes in BA 10, 46 and 9 in frontal lobes; BA 21, 37, 22 and 42 in temporal lobes; BA 43 and 3 in parietal lobes; and BA 19 in the occipital lobe. These findings support the view that individual differences in gray and white matter volumes, in a relatively small number of areas distributed throughout the brain, account for considerable variance in individual differences in general intelligence” (Haier et al., 2004).

Regarding the parts of the brain that are related to cognition, Barbey et al. (2012) state that “these structures are located primarily within the left prefrontal cortex (behind the forehead), left temporal cortex (behind the ear) and left parietal cortex (at the top rear of the head) and in white matter association tracts that connect them”. “By analyzing 182 patients with focal brain damage using voxel-based lesion–symptom mapping and comparing their cognitive abilities with those of patients in whom the same structures were intact, the researchers were able to identify brain regions essential to specific cognitive functions, and those structures that contribute significantly to intelligence”, Barbey said. Patients with Alzheimer’s disease show smaller gray matter volumes in the medial temporal lobe than do age-matched elderly individuals (Busatto et al., 2008; Taki et al., 2011). Decreased global cortical cortex and orbitofrontal cortex have been associated with diminished attention and executive function (Zimmerman et al., 2006; Kramer et al., 2007). Narr et al. (2007) emphasize that “neuropsychological patients with focal brain lesions provide a rare opportunity to study the mechanisms underlying general intelligence and executive functions, supporting the investigation of lesion-deficit associations”. In particular, the pattern of findings shows that high-level cognitive processes fundamentally depend on the interregional communication between frontal and parietal cortex (Barbey et al., 2012). Reinforcing the aforementioned, Damasio (2000) points out that one way to analyze how a part of the brain affects consciousness or in this case general intelligence is to analyze the behavior of individuals who have specific damage in those areas of the brain. Only when parts of the brain cease to function may one learn and recognize one’s biological purposes.

Cole et al. (2012) mark that control of thought and behavior is fundamental to human intelligence and a frontoparietal brain network implements such cognitive control across diverse contexts. Vakhtin et al. (2014) posit that the “refinement of localization of intelligence in the human brain is converging onto a distributed network that broadly conforms to the Parieto-Frontal Integration Theory (P-FIT)”. Colom et al. (2006) also state that the P-FIT nominates several areas distributed throughout the brain as relevant for intelligence. The P-FIT model is consistent with the generalist genes hypothesis mentioned by Kovas and Plomin (2006). Jung and Haier (2007) posit that not all these brain areas are equally necessary in all individuals for intelligence. They predict that discrete brain regions of the dorsolateral prefrontal cortex (BAs 9, 45, 46 and 47) and the parietal cortex (BAs 7 and 40) may be key for the core of general intelligence. Narr et al. (2007) state that “variations in prefrontal and posterior temporal cortical thickness are particularly linked with intellectual ability”. Given that cortical thickness generally relates with the number of neurons, it is regularly taken as indicative of the cognitive abilities of an individual.

### Molecular Genetics and Intelligence

Studies that seek to find quantitative trait loci have been relatively unsuccessful because of the great number of genes that may affect brain processes (Luciano et al., 2006). A wide-scale effort to identify genes that influence IQ was initiated by Plomin et al. (1995). They examined the association between 100 DNA markers in/near genes relevant to neural functioning in high and low IQ groups (Plomin et al., 1995).

In recent years, there has been a breakthrough in the genetic study of human intelligence because five whole-genome linkage scans have been published and all of them converged on several chromosomal regions that are important to intelligence. “Finding genes brings us closer to an understanding of the neurophysiological basis of human intelligence” (Posthuma and De Geus, 2006).

Two main strategies were followed to identify genes related to variations in cognition: genetic linkage analysis (Lincoln and Lander, 1992; O’Connell and Weeks, 1998; Posthuma and De Geus, 2006; Plomin and Deary, 2015) and candidate-gene association studies (Chorney et al., 1998; Kwon and Goate, 2000; Tabor et al., 2002; Zhu and Zhao, 2007).

Linkage is the tendency for genetic markers (DNA sequences or genes) to be inherited together because of their location near one another on the same chromosome. The nearer two genes are on a chromosome, the lower the chance of recombination is between them. Linkage analysis serves as a way of gene-hunting and genetic testing, which is both useful and used to identify individuals with a particular trait, such as cognitive ability or general learning disability.

The first whole-genome linkage scan for intelligence was published by Posthuma and De Geus (2006). Results derived from the studies of two samples (150 Dutch sibling pairs and 475 Australian sibling pairs) show that there are two areas of significant linkage to intelligence, chromosomes 2q and 6p, and several areas of suggestive linkage (chromosomes 4p, 7q, 20p and 21p). “The chromosome-2 area has been implicated in linkage scans for autism and dyslexia, while the chromosome-6 area is the main linkage area for reading ability and dyslexia” (Posthuma and De Geus, 2006). Two other studies also confirm the link between chromosomes 2 and 6 and cognition (Luciano et al., 2006; Wainwright et al., 2006). The findings (data from 210 families were analyzed) suggest that the genes on chromosome 2, which have a broad influence on a variety of cognitive abilities, influence the variation in general academic achievement (Wainwright et al., 2006). The CHRM2 gene could also be correlated with slight differences in performance IQ scores. This is not a gene for intelligence. It is a gene that is involved in some kinds of brain processing, and specific alterations in the gene appear to influence IQ. “But this one gene alone isn’t going to make the difference between whether a person is a genius or has below-average intelligence” (Dick et al., 2007). Finally, it is good to point out that Posthuma and De Geus (2006) presented an ideogram (chromosome map) of the human genome, showing which regions of chromosomes are likely to contain genes for intelligence. This ideogram is based on the five linkage studies conducted to date.

The candidate-gene association approach, on the other hand, is based on studies focused on associations between genetic variation within pre-specified genes of interest and phenotypes or disease states. Variation in the alleles is measured and tested for association with intelligence. The candidate gene is largely limited by its reliance on the priori knowledge about biological, physiological, or functional relevance of possible candidates. While the identification of candidate genes involved in genetic traits continues to be a challenge, significant progress in this subject has been achieved during the last few years (Zhu and Zhao, 2007).

Chorney et al. (1998) published the first candidate-gene association study in children. The conjunction results of two samples of Caucasians (*N* = 51, average IQ = 136136; *N* = 52 average IQ = 156) showed robust positive correlations between the gene insulin-like growth factor-2 receptor (IGF2R) on chromosome 6, region 6q26 and cognitive results. In the first study, “a DNA marker in the gene for IGF2R on Chromosome 6 yielded a significantly greater frequency of a particular form of the gene (allele) in a high-g group (0.303; average IQ = 136, *N* = 51) than in a control group (0.156; average IQ = 103, *N* = 51). This association was replicated in an extremely-high-g group (all estimated IQs > 160, *N* = 52) as compared with an independent control group (average IQ = 101, *N* = 50), with allelic frequencies of 0.340 and 0.169, respectively” (Chorney et al., 1998). They concluded that the IGF2R gene is associated with high g. Four years later, however, they conducted a replication analysis for a new sample that was bigger than the two previously reported samples combined. They concluded that the IGF2R gene is not associated with high g (Hill et al., 2002). “This finding is part of a long list of reported associations that have failed to be replicated (Cardon and Bell, 2001), and those associations that have shown some replication, such as dopamine gene associations with hyperactivity (Thapar, 2003), have not done so consistently” (Hill et al., 2002).

Genes associated with mental retardation (MR) are another source of candidates. Inlow and Restifo (2004) identified 282 genes related to MR in a sample of 1010 Online Mendelian Inheritance in Man (OMIM) database entries. They estimate that hundreds more MR genes remain to be identified.

More recently, Desrivières et al. (2015) conducted a large-scale association study of 1583 adolescents to identify genes affecting cortical thickness. They analyzed more than 54,000 genetic variants possibly involved in brain development and found that, on average, teenagers with a particular gene variant, rs7171755, associated with a thinner cortex in the left hemisphere (*P* = 1.12 × 10^−7^), particularly in the frontal and temporal lobes, were the ones who did not perform as well on tests of intellectual ability. Localized effects of these single-nucleotide polymorphisms on cortical thickness affected verbal and nonverbal intellectual abilities differently.

Desrivières says that the genetic variation they found is “linked to synaptic plasticity and affects a gene known as NPTN, which encodes a protein acting on neuronal synapses and therefore affects how brain cells communicate. This may help neuroscientists understand what happens at a neuronal level in certain forms of intellectual impairments, where the ability of the neurons to communicate effectively is somehow compromised”. Desrivières suggests the left hemisphere may be more sensitive to the effects of NPTN mutations, and that some differences in intellectual ability are due to decreased NPTN function in particular regions of the left-brain hemisphere.

Finally, it is good to point out that the candidate-gene approach has been criticized due to the lack of replication of results as well as its limited ability to include all possible causative genes (Tabor et al., 2002). Until now, only one association is known between genes and intelligence. This is the gene CHRM2, cholinergic receptor muscarinic 2, located in chromosome 7, region 7q33, replicated in different candidate-gene studies (Comings et al., 2003; Jones et al., 2004; Dick et al., 2007). The gene contributes 1%–2% of the variance in full-scale IQ.

## The Co-Action Between Genetic Factors, Environment and Cognition

Genetic factors have a significant contribution in defining brain structure and cognition. In particular, some studies show that cortical thickness is heritable and closely correlates with intellectual ability in normally developing children and adolescents (Hulshoff Pol et al., 2006; Panizzon et al., 2009; Haworth et al., 2010; Blokland et al., 2012; Chen et al., 2012; Eyler et al., 2012; van Soelen et al., 2012; Trzaskowski et al., 2014; Desrivières et al., 2015). Other studies report high heritability of gray-matter volume in several cortical regions using voxel-based MRI techniques (Thompson et al., 2001; Posthuma et al., 2002; Bohlken et al., 2016). In addition, Thompson et al. (2001) published “three-dimensional maps revealing how brain structure is influenced by individual genetic differences. A genetic continuum was detected in which brain structure was increasingly similar in subjects with increasing genetic affinity. Genetic factors significantly influenced cortical structure in Broca’s and Wernicke’s language areas, as well as frontal brain regions (r2MZ > 0.8, *p* < 0.05)”. Bohlken et al. (2016) also state that genetic factors implicated in intelligence and gray matter are found in specific regions, pertaining primarily to the medial/superior frontal, occipital and parahippocampal cortices and the thalamus.

Gray matter correlated substantially with general intelligence, or “g”. Deary et al. (2009) state that the heritability of g is substantial. “It increases from a low value of about 30% in early childhood, to well over 50% in adulthood, which continues into old age. Despite this, there is still almost no replicated evidence concerning the individual genes, which have variants that contribute to intelligence differences”. As Maher (2008) says, intelligence is not unusual in the difficulties it has found in trying to identify the genes responsible for its high heritability.

A highly connected brain network is surely the key to the neural processes that give rise to intelligence. But, the extent to which brain networks linked to intelligence are shaped through genes and environment is not known (Bohlken et al., 2016).

### Epigenetic and Brain Development

Epigenetic mechanisms typically involve heritable changes in chromatin structure, which, in turn, influence gene expression without modifying actual DNA sequence. “The expansion of the brain after birth is caused by the growth of synapses and cortical interconnections that are dependent on nutritional conditions and the environment” (Cairó, 2011). It is clear that the process of brain development is vulnerable to adverse environmental conditions (Gittleman, 1986; Leonard and Robertson, 1994; Rao and Jacobson, 2005; Lister et al., 2013; Bale, 2015). For instance, the inhabitants of sub-Saharan Africa, the area of the continent of Africa that lies south of the Sahara desert, have a small cranial capacity, but undoubtedly poor environmental conditions, minimal health care, bad nutrition of most individuals and poor government policy influence normal brain growth. In this case the poor environmental conditions are the ones that mainly determine the IQ of an individual.

Significantly, diet is linked to brain size in primates. A diet rich in meat lead to bigger brains (Foley et al., 1991) and human evolution (Mann, 2000). Beals et al. (1984) also state that cranial morphology is a reflection of thermoregulation, and changes in head shape may increase or decrease cranial volume. “It is much easier to keep a small head cooler than a large one” (Cairó, 2011). From this perspective, a small head is convenient in hot regions (south) while in geographic areas with cold environments (north) a large head is an advantage. The same seems to happen with the volume of the eyes. This does not necessarily means that people from the north is smarter than people from the south. Beals et al. (1984) reported a correlation of 0.62 between cranial capacity and distance from the equator. Pearce and Dunbar (2012) say: “As you move away from the equator, there’s less and less light available, so humans have had to evolve bigger and bigger eyes. Their brains also need to be bigger to deal with the extra visual input”. Jensen (1998) reasoned that “natural selection would favor a smaller head with a less spherical shape because of better heat dissipation in hot climates”.

### Brain Plasticity

The brain has amazing plasticity and it is particularly important during adolescence, when both hormonal and social environments change dramatically (Dahl, 2004; Desrivières et al., 2015). The growth of white matter during human adolescence increases linearly, but follows a strikingly different trajectory in girls and boys. It increases with age, slightly in girls and steeply in boys (De Bellis et al., 2001; Lenroot et al., 2007; Perrin et al., 2008).

Research now shows that when a new cognitive or motor skill is learned the structure of the human brain, as well as its functional organization, changes (Draganski et al., 2004; Driemeyer et al., 2008). Particularly, an increase of gray matter is observed, what is known as neuroplasticity or cortical re-mapping. “While functional changes can be observed and studied with non-invasive techniques structural changes require the use of invasive histological methods” (Cairó, 2011).

Fluid intelligence is considered one of the most important factors in learning. With more training, our fluid intelligence improves. “An increase in fluid intelligence is directly related to the changes in the structure of the human brain and its functional organization” (Cairó, 2011). Jaeggi et al. (2008) points out that “the gain in fluid intelligence is strictly related to training, not to pre-existing individual differences in intelligence or working memory”.

Gaser and Schlaug (2003) “found more gray matter in some motor, auditory, visual-spatial brain regions when comparing professional musicians with a matched group of amateur musicians and non-musicians”. Different experiments show that brain plasticity on a structural level in human beings has been detected even after a week of training, although changes over a longer period of time are still a subject for debate (Maguire et al., 2000; May et al., 2006; Vestergaard-Poulsen et al., 2009). Even playing a commercial video game like Super Mario induces gray matter changes (Kühn et al., 2014).

Even though it is thought that all areas of the brain are plastic, results show that areas of the brain, such as hippocampus, dentate gyrus and cerebellum, are actually highly plastic. This means that new neurons can be produced even in adulthood.

### Gene-Environment Interplay

“Cognitive development and functioning can be significantly affected by the following factors: environmental influences, including geographic area (iodine deficiency, lead exposure, climate, logographic writing systems); nutrition (insufficient iron, insufficient meat); socioeconomic status (poverty); physical and psychological disorder (violence, extreme aggression, societal disruption); education (lack of motivation, lack of confidence that learning certain skills will bring about a change in one’s life); culture, insufficient intellectual stimulation (children living in orphanages); or the combination of any of the above-mentioned factors” (Cairó, 2011). Even variations in parental care can lead to individual differences in the expression of genes. Moffitt (2005) also states that “emerging evidence about gene-environment interactions suggests that environmental risks can have a stronger effect on people within genetically vulnerable segments of the population than previously thought”. These factors can also alter the effects of genes and be considered the primary reason for the difference of IQ among ethnic groups.

People differ of course not only in dress, habits, types of occupations, moral and political attitudes, but also in their abilities and achievements (Vernon, 2014). The question could be why? And the answer could be because brains are different, they develop in a different environment, and therefore, people think and reason in different ways. We have always been interested in knowing why some brains are more efficient than others, and now the answers are beginning to see the light with the recent advances in neuroscience and cognitive science. Most parts and tissues of the brain have a large genetic load. But the environmental factors can significantly affect the development of the brain-connected network, which is the key for the neural processes that give rise to intelligence. In short, cognition is a combination of internal and external factors, both of which can vary greatly from person to person. It is estimated that genes contribute about 20%–40% in the discrepancy of intelligence in childhood and about 80% in old age when the environment is no longer an important factor.

As babies, our neuronal associations are totally undifferentiated. “In order for persons to develop certain intellectual abilities, they need to have the appropriate environmental stimuli during childhood, before the critical period for adapting their neuronal connections ends (at the age of 16)” (Garlick, 2002). He states that the critical period effect is a result of the manner by which intellectual abilities are acquired—that changes in neuronal connections inhibit or prevent possible future changes.

There are different types of experiments demonstrating the above. Skeels et al. (1938) conducted an interesting study of early intellectual stimulation in the state of Iowa in the US, with 25 underprivileged children living in an overcrowded (600 children were in residence at the beginning of the project) and understaffed orphanage. Thirteen babies, who were 19 months old and with an average IQ of 64, were transferred to the Glenwood State School for adult woman with learning disabilities, while the other 12 who remained in the orphanage had an average IQ of 87. “After 18 months, the babies who received the finest care, love and attention from their teachers were tested again and the average IQ jumped to 93” (Cairó, 2011). Eleven of the 13 girls were given up for adoption. Thirty months later, when the IQ of these 11 girls was evaluated again, it jumped to an average of 101 points. On the other hand, the children who remained now had an average IQ of 66. The research studies demonstrated the remarkable effect of the environment on intelligence. An enhanced learning environment, where children are stimulated and motivated, can significantly expand IQ whereas a deprived learning environment can lead to a decrease in IQ.

Hart and Risley (2003) conducted a longitudinal study with children who were 4 years old (42 families, 42 children) and were growing up in poor families in Kansas City, in the US. They found that these children typically heard a total of 32 million fewer spoken words than those whose parents are professionals. “The average child on welfare heard half as many words per hour (616 words per hour) as the average working-class child (1251 words per hour) and less than one-third that of the average child in a professional family (2153 words per hour)” (Hart and Risley, 2003). That language gap translates directly into stunted academic trajectories. At the same time, they found that they could easily increase the size of the children’s vocabularies by teaching them new words.

Another study was conducted in France with a group of children between 4 and 6 years of age who were adopted. “Those children had little going for them. Their I.Q.’s averaged 77, putting them near retardation. Most were abused or neglected as infants, then shunted from one foster home or institution to another” (Kirp, 2006). After 9 years these same youngsters were tested again. Those children adopted by farmers or laborers had an IQ of 85.5; children who were placed in middle-class families had an IQ of 92; and those in more affluent families reached an IQ of 98. A home environment that provides easy access to intellectual knowledge “can provide the mental stimulation needed for genes to build the brain circuitry for intelligence”, as Turkheimer says. Again, the experience and quality of life of these three groups are what made the main difference.

More recently, Caspi et al. (2003) explored the roles of a variation in a gene that alters serotonin levels and exposure to stressful life events across a 20-year period in determining the risk of depression (Champagne and Mashoodh, 2009). “Individuals with one or two copies of the short allele of the 5-HT T promoter polymorphism exhibited more depressive symptoms, diagnosable depression, and suicidality in relation to stressful life events than individuals homozygous for the long allele. This epidemiological study thus provides evidence of a gene-by-environment interaction, in which an individual’s response to environmental challenges is moderated by his or her genetic makeup” (Caspi et al., 2003).

Monozygotic twins’ studies also provide important comprehension into epigenetic effects in humans. Monozygotic twins do not differ genetically, but they have the potential to differ in terms of how, when, and where genes are expressed (Kramer, 2005). Fraga et al. (2005) found in a sample of 80 Caucasian twins from Spain (30 male, 50 female, mean age 30.6, range 3–74 years) that, “although twins are epigenetically indistinguishable during the early years of life, older monozygous twins exhibited striking differences in their overall content and genomic distribution of 5-methylcytosine DNA and histone acetylation, affecting their gene-expression portrait”. Their study reveals that the patterns of epigenetic modifications in monozygotic twins diverge as they become older. DNA *methylation* and histone modifications (which can be dependent on one another) store epigenetic information that controls heritable states of gene expressions, protein function and RNA processing. DNA methylation can also keep inactive a chromosome and produce different disorders including cancer (Ehrlich, 2002; Das and Singal, 2004).

These examples show that genetics and environment cannot be evaluated as two separated spheres. “Genetic instructions are not translated directly into phenotypic traits, rather they are modified potentially at two levels: the transcription process wherein the messenger RNA is produced, and translation when protein synthesis occurs. The interplay of genetic and environmental factors determines the final product of gene expressions” (Kramer, 2005). In a chaotic environment, a child’s genetic potential does not have a chance to be expressed (Turkheimer et al., 2003). “On the other hand, properly stimulated and motivated children may be able to change the brain structure and its functional organization and surpass the genetic potential” (Cairó, 2011). “Results demonstrate that the proportions of IQ variance attributable to genes and environment vary nonlinearly with socioeconomic status. The models suggest that in impoverished families, 60% of the variance in IQ is accounted for by the shared environment, and the influence of genes is close to zero; in affluent families, the result is almost exactly the opposite” (Turkheimer et al., 2003). The appropriate environment not only leads to increasing the complexity of the brain connection but also to increasing the brain mass and volume (Kolb and Whishaw, 1998; Garlick, 2002). All these studies show that: “it is no longer a matter of whether the environment matters but when and how it matters. And poverty, quite clearly, is an important part of the answer” (Kirp, 2006).

Finally, Rutter (2010) outlines well the different forms of co-action between genes and environment. He says that “environments cannot alter gene sequences, but genetic effects are dependent on the expression of genes. Both environmental influences and chance variations have been shown to influence this process”.

## Conclusion

The results of this review show that the complexity of cellular and molecular organization of neural connections actually determines the computational capacity of the brain. The consistency of new results across the literature has revealed that individuals with more efficient brain networks have a higher intelligence. Intelligence is genetically represented in a spatially distributed, but densely connected network of gray matter regions, allowing the high capacity infrastructure thought necessary for this complex trait to emerge (Bohlken et al., 2016). Research also clearly notes that experience plays an indisputable role in the intellect of individuals.

The research key findings show that while genotypes are responsible for defining the theoretical limits of intelligence, what is primarily responsible for determining whether those limits are reached or exceeded is experience (environmental influence). Everything indicates that experience makes the great difference, and therefore, we contend that the gene-environment interplay defines the intelligent quotient of an individual.

How are brain structure and genes mapped on behavior and intelligence? We don’t know yet. Many questions have yet to be answered, and numerous issues obviously require much further research.

## Funding

This work has been supported by Asociación Mexicana de Cultura A.C.

## Conflict of Interest Statement

The author declares that the research was conducted in the absence of any commercial or financial relationships that could be construed as a potential conflict of interest.
